# ﻿Application of RAPD markers for *Cuscuta* species identification and biodiversity

**DOI:** 10.3897/phytokeys.265.152696

**Published:** 2025-10-14

**Authors:** Denitsa Teofanova, Kalina Pachedjieva, Anita Tosheva, Bianka Marinova, Stefan Savov, Martin Savov, Tzvetelina Zagorcheva, Lyuben Zagorchev

**Affiliations:** 1 Department of Biochemistry, Faculty of Biology, Sofia University “St. Kliment Ohridski”, 8 Dragan Tsankov blvd., 1164, Sofia, Bulgaria Sofia University “St. Kliment Ohridski” Sofia Bulgaria; 2 AgroBioInstitute, Agricultural Academy, 8 Dragan Tsankov blvd., 1164, Sofia, Bulgaria AgroBioInstitute, Agricultural Academy Sofia Bulgaria; 3 Research & Development & Innovation Consortium, 111 Tsarigradsko shose blvd., 1784, Sofia, Bulgaria Research & Development & Innovation Consortium Sofia Bulgaria

**Keywords:** Dodder, genetic diversity, molecular markers, parasitic plants, RAPD markers

## Abstract

*Cuscuta* species (dodders) are parasitic plants that harm both native and cultivated flora, presenting significant ecological and economic challenges. This study employs random amplified polymorphic DNA (RAPD) markers to evaluate genetic variation among 70 specimens collected from various localities across Bulgaria. The findings reveal notable differences in RAPD profiles between species, indicating that RAPD markers are effective for species identification, particularly when reproductive organs are absent. While *C. campestris* exhibited a relatively uniform genetic profile across different populations, native species showed greater genetic heterogeneity, likely due to their development in diverse habitats. Geographical patterns were also observed in the genetic clustering of *C. campestris*, although seed dispersal mechanisms appeared to play a larger role in shaping genetic diversity than geographic distribution alone. This research highlights the potential of RAPD markers for improving species identification and for understanding the genetic dynamics of *Cuscuta* populations, which is crucial for managing their spread and minimizing their agricultural impact.

## ﻿Introduction

*Cuscuta*, commonly known as dodders, is a genus of parasitic plants belonging to the family Convolvulaceae. These species are stem holoparasites, parasitizing the above-ground host tissues and relying entirely on host plants for water and nutrients (Dawson et al. 1994). The genus comprises around 200 species worldwide, many of which are considered significant agricultural pests due to their detrimental effects on various crops (Parker 2012). In Bulgaria, *Cuscuta* species are widely distributed across diverse habitats, including agricultural lands, grasslands, and natural ecosystems. Their presence poses ecological and economic challenges, affecting both native flora and cultivated plants (Teofanova et al. 2022). A total of nine to ten species have been recorded in the country (Assyov and Petrova 2012; Stoyanov et al. 2021), with variable distribution. The most common and usually associated with anthropogenic impact is the introduced North American species *Cuscuta campestris* Yunck., which is also the most widely distributed species in the genus worldwide (Parker 2012). Other common species in the country include *C. epithymum*, *C. europaea*, and *C. approximata*. Besides species diversity and distribution, however, little is known about the population structure and genetic diversity within the recorded species. Such data would be of significant interest to better understand the spread of the harmful *C. campestris*, as well as to connect the genetic background to host range, preference, and geographic distribution.

Traditional morphological identification of *Cuscuta* species is often complicated due to their reduced vegetative structures and close similarity of reproductive organs (Stefanović et al. 2007). Identification when flowers or seeds are not present is almost impossible. This challenge has led to the increasing use of molecular markers in species identification and population studies. Species identification is often based on chloroplast genome sequences (Park et al. 2019) or the ITS region of nuclear ribosomal DNA and ribulose bisphosphate carboxylase large subunit (*rbcL*) (Keskin et al. 2017; Masanga et al. 2022). Besides species identification, both ITS and *rbcL* proved efficient in genetic diversity studies. Population studies within the genus, however, are relatively scarce. These include the employment of molecular markers, such as ISSR (Tajdoost et al. 2013; Alikelayeh et al. 2014), RAPD (Khan et al. 2010; Noshad et al. 2021), and SCAR (Abdin et al. 2012). Most authors concluded that the mechanism of dispersal—by seeds and vegetative propagation by vines—affects the genetic structure of *Cuscuta* populations. However, the findings were somehow contradictory, as Tajdoost (Tajdoost et al. 2013) reported low diversity within populations and high diversity among populations of *C. campestris*, while Masanga (Masanga et al. 2022) reported significant mixing among populations.

No such studies were reported for Bulgaria. Among the various molecular approaches, Random Amplified Polymorphic DNA (RAPD) markers have proven to be particularly effective in assessing genetic variation in plants (Tingey et al. 1994). RAPD markers are dominant markers that do not require prior genetic information, making them highly suitable for studies on non-model organisms and species with limited genomic resources, which is especially valuable in a genus like *Cuscuta*, where plastid genomes are readily available (McNeal et al. 2007; Pan et al. 2023), but nuclear genome sequences are scarce.

This study aims to explore the distribution of *Cuscuta* species in Bulgaria and evaluate their genetic diversity using RAPD markers. By employing molecular techniques, we can enhance species identification accuracy, assess genetic variation among populations, and gain valuable insights into the geographic distribution and host preferences of these parasites. Understanding the genetic structure of *Cuscuta* populations will not only aid in taxonomic classification but also support efforts in controlling their spread and mitigating their impact on agriculture and biodiversity.

## ﻿Material and methods

### ﻿Plant material

**Figure 1.**
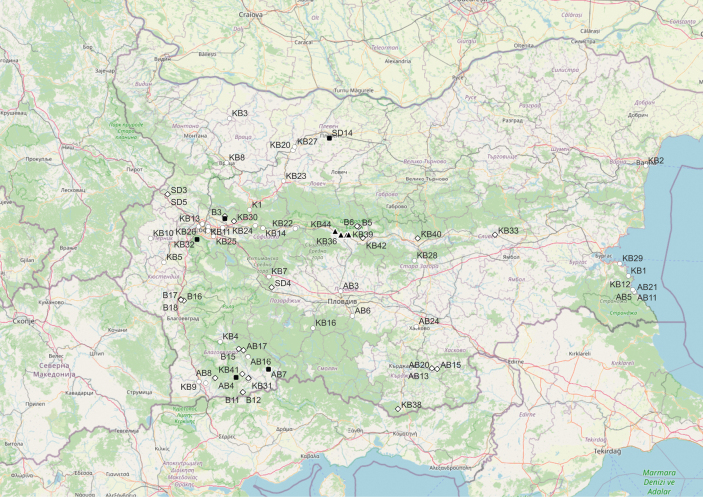
Geographic distribution of the studied specimens of *C. approximata* (black triangle), *C. campestris* (white circle), *C. epithymum* (white diamond), and *C. europaea* (black square).

A total of 75 *Cuscuta* specimens from different localities in Bulgaria, collected between 2017 and 2024 and stored at −80 °C, were used in the present experiment. The representation of each species is as follows: *C. campestris* – 39 specimens, *C. approximata* – 5 specimens, *C. epithymum* – 26 specimens, and *C. europaea* – 5 specimens. All specimens are listed in Suppl. material 1 and shown as a distribution in the country in Fig. 1. A high-resolution version of Fig. 1 is also provided as Suppl. material 4. The map was generated with QGIS Desktop version 3.34.8 (QGIS Development Team 2025).

### ﻿DNA isolation and separation

For DNA isolation, vegetative material was ground in liquid nitrogen, and total DNA was isolated using the GeneMATRIX Plant & Fungi DNA Purification Kit (EURX, Gdansk, Poland), following the manufacturer’s instructions. The obtained DNA concentration was determined spectrophotometrically with a NanoDrop 2000c (Waltham, MA, USA) at A260. The purity was assessed based on the absorption ratio at λ = 260/280 nm. Isolated total DNA and PCR products were separated on a 1% agarose gel and visualized using CSL Runsafe (Cleaver Scientific Ltd., Rugby, UK) under UV light. To determine fragment sizes, DirectLoad Wide Range DNA Marker (Sigma Aldrich, Saint Louis, MI, USA) was used.

### ﻿RAPD analysis

Random amplified polymorphic DNA (RAPD) analysis was conducted using a single primer serving simultaneously as both forward and reverse, binding to different DNA sites. The PCR reaction conditions were as follows: (1) initial denaturation at 94 °C for 5 min; (2) 40 cycles: denaturation at 94 °C for 1 min, primer annealing at 36 °C for 1 min, extension at 72 °C for 1 min; and (3) final extension at 72 °C for 7 min. All amplifications were performed in triplicate to ensure the reproducibility of the obtained fragments. Five decamer primers were used: OPA-03 (5′-AGTCAGCCAC-3′), OPA-07 (5′-GAAACGGGTG-3′), OPB-17A (5′-GACCGCTTGT-3′) (Rai et al. 2015), OPB-17B (5′-AGGGAACGAG-3′), and OPAL-20 (5′-AGGAGTCGGA-3′) (Elsiddig et al. 2018). Amplification was performed using KAPA PROBE FAST Master Mix (Merck) on a Techne Thermal Cycler. Amplification products were separated via agarose gel electrophoresis, and electropherograms were analyzed using GelAnalyzer version 23.1.1 (Lazar et al. 2010). Fragment polymorphism was constructed manually as a binary data table: 1 for presence, 0 for absence, and NA for non-amplified samples (Suppl. material 2). Only clear bands were considered. The binary matrix was further corrected (Suppl. material 3) by removing the data for the primer with the lowest efficiency and the data for samples that were not amplified by some of the primers. The binary matrix was used to construct a UPGMA dendrogram by the neighbor-joining cluster method (Jaccard coefficient), using PAST software version 5.1 (Hammer et al. 2001). Additionally, principal coordinate analysis of the clustering, based on floristic region, was performed using R version 4.5.1 (R Core Team 2025), with the vegan (Oksanen et al. 2020), ggplot2 (Wickham 2016), and dplyr (Wickham et al. 2023) packages.

**Table 1.** Efficiency of RAPD primers. s – number of samples; as – number of amplified samples; e – efficiency in percentage, calculated as e = (as/s) × 100.

Primer	Cuscuta campestris			Cuscuta epithymum			Cuscuta europaea			Cuscuta approximata			Average efficiency
	s (n)	as (n)	e (%)	s (n)	as (n)	e (%)	s (n)	as (n)	e (%)	s (n)	as (n)	e (%)	
OPA-03	39	37	94,9	26	23	88,5	5	5	100,0	5	5	100,0	95,8
OPA-07	39	32	82,1	26	21	80,8	5	4	80,0	5	4	80,0	80,7
OPB-17A	39	38	97,4	26	23	88,5	5	5	100,0	5	5	100,0	96,5
OPB-17B	39	30	76,9	26	17	65,4	5	3	60,0	5	1	20,0	55,6
OPAL-20	39	37	94,9	26	25	96,2	5	5	100,0	5	2	40,0	82,8

## ﻿Results and discussion

The five primer pairs were chosen based on their ability to amplify multiple fragments and their high percentage of polymorphic fragments in different *Cuscuta* specimens (Rai et al. 2015; Elsiddig et al. 2018). Electropherograms of the amplified RAPD fragments are provided as Suppl. material 5. Despite multiple repetitions, some of the primers failed to amplify certain samples, which is shown in Table 1. OPB-17B was notable for its low efficiency among all four *Cuscuta* species.

A summary of the observed fragments is provided in Table 2. The number of fragments varied significantly between species and between primer pairs, ranging from as low as 1 to as high as 15. Overall, the number of fragments was the lowest in *C. approximata*, with the exception of OPB-17A. *Cuscuta campestris* and *C. epithymum* gave the highest overall numbers. This outcome may be affected by the number of specimens tested within each species. The number of fragments, as well as the number of polymorphic fragments obtained, was in good agreement with published results for RAPD profiling of *Cuscuta* spp. (Khan et al. 2010; Kazemitabar et al. 2014; Elsiddig et al. 2018). It should be noted, however, that OPB-17B gave a relatively high number of fragments and almost 100% polymorphism, but its very low efficiency made it unsuitable in this particular analysis.

**Table 2.** Number of fragments observed for each species with each primer. Numbers in brackets represent polymorphic fragments. Average numbers were rounded up to an integer.

	C. campestris	C. epithymum	C. europaea	C. approximata	Average
OPA-03	7 (4)	8 (7)	7 (6)	6 (4)	7 (5)
OPA-07	12 (9)	15 (15)	8 (7)	5 (3)	10 (9)
OPB-17A	9 (4)	15 (15)	12 (12)	11 (10)	12 (10)
OPB-17B	13 (13)	11 (11)	7 (6)	1*	8 (8)
OPAL-20	13 (10)	8 (8)	9 (8)	7 (3)	9 (7)
Average	11 (8)	11 (11)	9 (8)	6 (4)	

**Figure 2.**
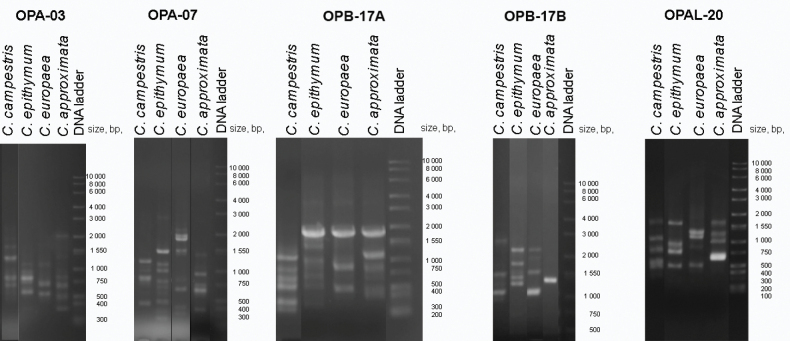
Indicative RAPD profiles of four *Cuscuta* species with five different primer pairs. Specimens with the highest number of amplified fragments were selected for each.

The results showed that substantial differences in the RAPD profiles may be established among different species (Fig. 2). The RAPD profiles (Suppl. material 5) were used for manual construction of a raw binary matrix (Suppl. material 2), with all fragments detected by the five primers as variables in columns. The binary matrix was further cleaned (Suppl. material 3) by removing the OPB-17B fragments and all samples that failed to be amplified even by one of the primers (designated as NA—not available in the raw binary matrix.

The pairwise genetic distance matrix, resulting from the binary matrix, is shown in Table 3. Considering the within-species diversity, with the exception of *Cuscuta campestris*, where large clusters of specimens demonstrated a uniform RAPD profile with a particular primer, the other three species were shown to be more heterogeneous—showing more than twofold higher genetic distance. Such results strongly suggest that RAPD molecular markers could be successfully applied for the species authentication of different *Cuscuta* spp., which is an important feature considering the difficulties in taxonomic determination due to similarities between species and in seasons when reproductive organs are not available (Stefanović et al. 2007). The greater heterogeneity among the three native species (Table 3) could also be explained by their independent development in diverse habitats and with diverse host availability. Such processes were previously observed in other *Cuscuta* species and, in extreme cases, may even lead to speciation (Costea et al. 2020). It should also be noted that both *C. europaea* and *C. epithymum* have several recognized subspecies (Barath and Csiky 2012; García 2024) and may not be highly invasive outside their natural range (García 2024). Limited seed dispersal and gene flow between populations, due to the intrinsic nature of reproduction of these weeds (Tajdoost et al. 2013), may also contribute to the high genetic variability. As for *C. campestris*, being an introduced species with seed dispersal mainly associated with anthropogenic activities (Costea et al. 2016), it could be suggested that distant populations may have similar genetic profiles due to a common source.

**Table 3.** Pairwise genetic distance matrix.

	C. campestris	C. approximata	C. epithymum	C. europaea
*C. campestris*	8,3			
*C. approximata*	41,3	15		
*C. epithymum*	44,8	31	16,5	
*C. europaea*	40,3	28,8	30,2	17,2

**Figure 3.**
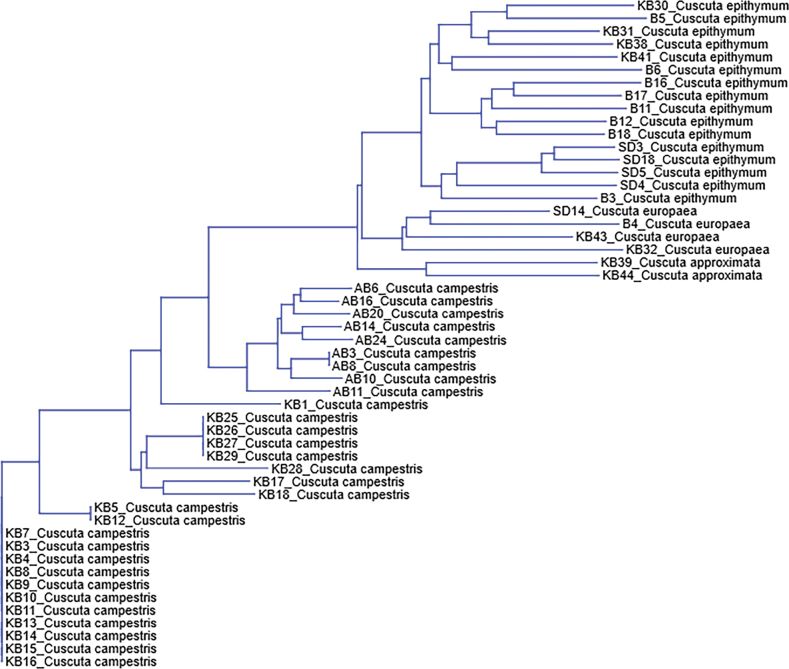
Neighbor-joining (Jaccard coefficient) phylogenetic tree of specimens of four *Cuscuta* species, based on a RAPD-built binary matrix.

The multivariate clustering, using the neighbor-joining (NJ) method with Jaccard genetic distance (Fig. 3), confirmed that *Cuscuta campestris* clustered separately from the other three species. The observed pattern confirmed previous results based on ITS sequence analysis (Teofanova et al. 2022), also supporting the genetic similarity of *C. approximata* to *C. europaea*, rather than to the phenotypically similar *C. epithymum*. However, all three species belong to the subgenus Cuscuta, unlike *C. campestris*, which belongs to the subgenus Grammica (Costea et al. 2015), explaining the higher genetic distance between them and the introduced species. The NJ tree also revealed higher genetic diversity within *C. epithymum* compared to *C. campestris*, where several clusters were formed by specimens with identical RAPD profiles.

**Figure 4.**
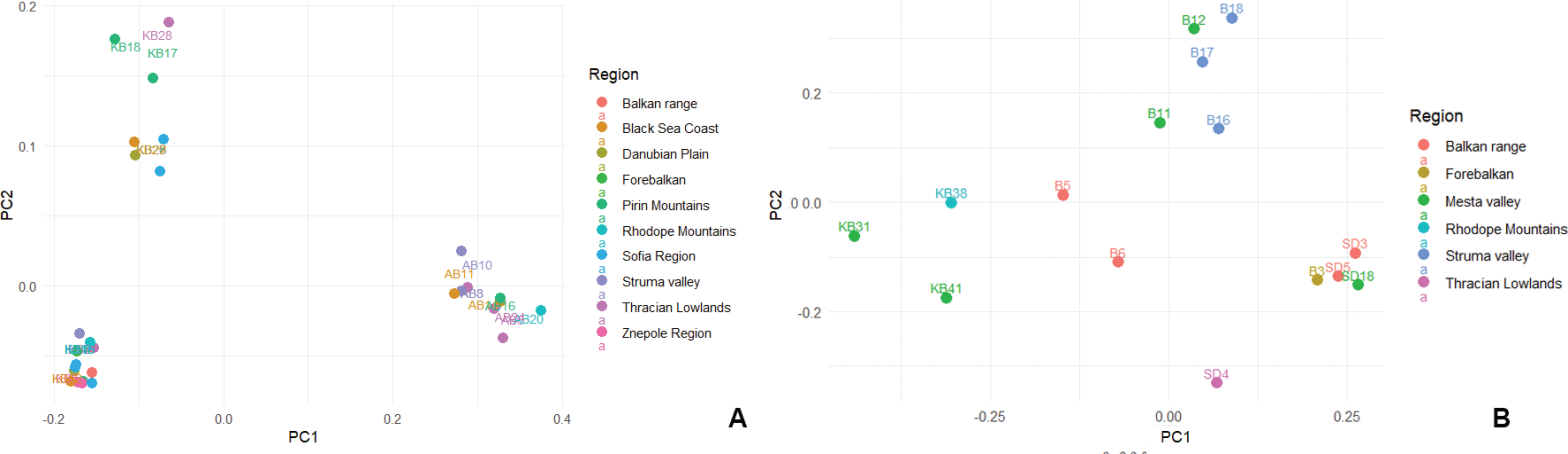
Principal coordinate analysis of *C. campestris* (A) and *C. epithymum* (B), based on RAPD profiles and grouped by floristic regions.

We further aimed to establish whether there is a geographic pattern in the RAPD profiles. The *C. campestris* and *C. epithymum* specimens were assigned to floristic regions (Assyov and Petrova 2012), and principal coordinate analysis was performed to determine whether specimens from the same floristic region clustered together (Fig. 4). In neither of the two species was good clustering of specimens from the same floristic region observed, suggesting that geographically close populations differ genetically. Although some closely located specimens were also genetically similar, especially in *C. epithymum*, in both species genetically similar specimens were located in distinct floristic regions, e.g., the Sofia region and the Black Sea Coast in *C. campestris*.

These results proved that despite some geographically related genetic similarities, the overall genetic diversity of *C. campestris* and *C. epithymum* in Bulgaria is not clearly related to distribution. This may be explained by the mode of seed dispersal, through contaminated commercial seed stocks of other species or soil seed banks (Olszewski 2019; Olszewski et al. 2020).

## ﻿Conclusion

This study demonstrates that RAPD markers are effective tools for identifying and assessing genetic diversity in *Cuscuta* species, especially when reproductive structures are unavailable. The results reveal significant genetic variation within native *Cuscuta* species and a relatively uniform genetic profile in *C. campestris*, likely influenced by anthropogenic seed dispersal. Understanding the genetic structure and distribution patterns of these parasites is crucial for improving species management and mitigating their impact on agriculture and biodiversity in Bulgaria.

## ﻿Additional information

### Use of AI

No use of AI was reported.

### Author ORCIDs

Denitsa Teofanova https://orcid.org/0000-0002-0467-485X

Kalina Pachedjieva https://orcid.org/0000-0001-5638-3467

Anita Tosheva https://orcid.org/0000-0002-4606-1672

Bianka Marinova https://orcid.org/0009-0008-6568-4294

Martin Savov https://orcid.org/0009-0008-7497-2909

Tzvetelina Zagorcheva https://orcid.org/0009-0007-5604-2719

Lyuben Zagorchev https://orcid.org/0000-0002-4765-1324

### Data availability

All of the data that support the findings of this study are available in the main text or Supplementary Information.

## Supplementary materials

Supplementary material 1 List of specimens Data type xlsx https://binary.pensoft.net/file/1437830This dataset is made available under the Open Database License (http://opendatacommons.org/licenses/odbl/1.0/). The Open Database License (ODbL) is a license agreement intended to allow users to freely share, modify, and use this Dataset while maintaining this same freedom for others, provided that the original source and author(s) are credited.Denitsa Teofanova, Kalina Pachedjieva, Anita Tosheva, Bianka Marinova, Stefan Savov, Martin Savov, Tzvetelina Zagorcheva, Lyuben Zagorchev

Supplementary material 2 Fragment polymorphism constructed as a binary data table Data type xlsx https://binary.pensoft.net/file/1437831This dataset is made available under the Open Database License (http://opendatacommons.org/licenses/odbl/1.0/). The Open Database License (ODbL) is a license agreement intended to allow users to freely share, modify, and use this Dataset while maintaining this same freedom for others, provided that the original source and author(s) are credited.Denitsa Teofanova, Kalina Pachedjieva, Anita Tosheva, Bianka Marinova, Stefan Savov, Martin Savov, Tzvetelina Zagorcheva, Lyuben Zagorchev

Supplementary material 3 The binary matrix Data type xlsx https://binary.pensoft.net/file/1437832This dataset is made available under the Open Database License (http://opendatacommons.org/licenses/odbl/1.0/). The Open Database License (ODbL) is a license agreement intended to allow users to freely share, modify, and use this Dataset while maintaining this same freedom for others, provided that the original source and author(s) are credited.Denitsa Teofanova, Kalina Pachedjieva, Anita Tosheva, Bianka Marinova, Stefan Savov, Martin Savov, Tzvetelina Zagorcheva, Lyuben Zagorchev

Supplementary material 4 A high-resolution version of Fig. 1 Data type tif https://binary.pensoft.net/file/1437833This dataset is made available under the Open Database License (http://opendatacommons.org/licenses/odbl/1.0/). The Open Database License (ODbL) is a license agreement intended to allow users to freely share, modify, and use this Dataset while maintaining this same freedom for others, provided that the original source and author(s) are credited.Denitsa Teofanova, Kalina Pachedjieva, Anita Tosheva, Bianka Marinova, Stefan Savov, Martin Savov, Tzvetelina Zagorcheva, Lyuben Zagorchev

Supplementary material 5 Electropherograms of the amplified RAPD fragments Data type pdf https://binary.pensoft.net/file/1437834This dataset is made available under the Open Database License (http://opendatacommons.org/licenses/odbl/1.0/). The Open Database License (ODbL) is a license agreement intended to allow users to freely share, modify, and use this Dataset while maintaining this same freedom for others, provided that the original source and author(s) are credited.Denitsa Teofanova, Kalina Pachedjieva, Anita Tosheva, Bianka Marinova, Stefan Savov, Martin Savov, Tzvetelina Zagorcheva, Lyuben Zagorchev
