# Supplementary material for: ﻿Application of RAPD markers for Cuscuta species identification and biodiversity
Source: PhytoKeys. 2025 Oct 14;265:1–12. doi: 10.3897/phytokeys.265.152696 (PMC12541467; doi:10.3897/phytokeys.265.152696)
Supplement: Supplementary material 5 — Electropherograms of the amplified RAPD fragments [file phytokeys-265-001_article-152696__-s005.pdf]

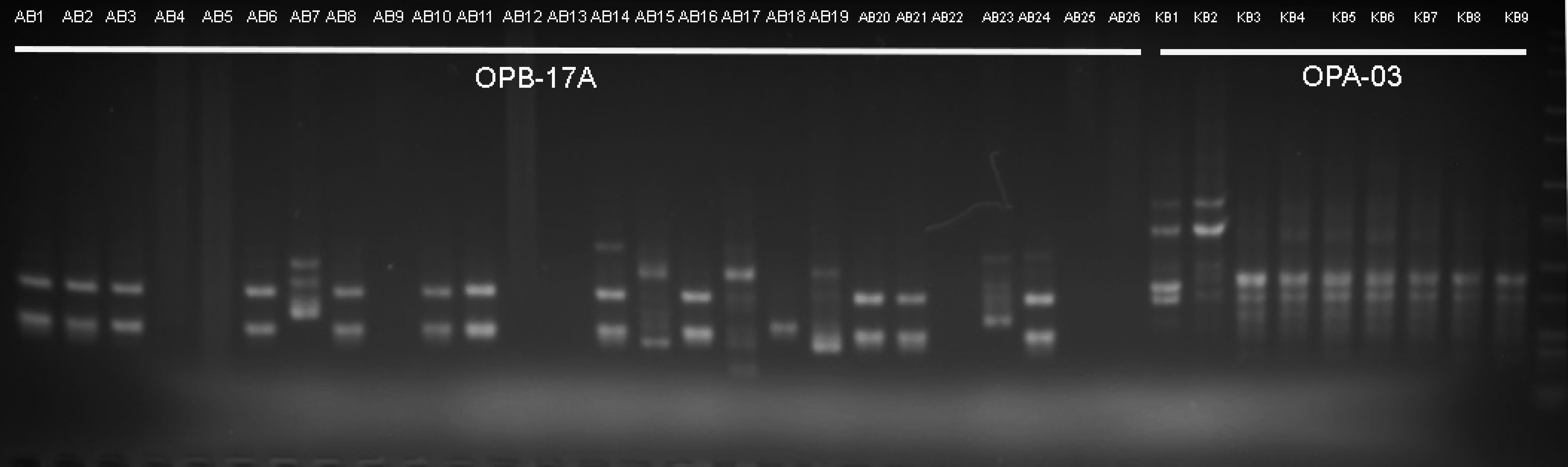
AB1 AB2 AB3 AB4 AB5 AB6 AB7 AB8 AB9 AB10 AB11 AB12 AB13 AB14 AB15 AB16 AB17 AB18 AB19 AB20 AB21 AB22 AB23 AB24 AB25 AB26 KB1 KB2 KB3 KB4 KB5 KB6 KB7 KB8 KB9
OPB-17A
OPA-03

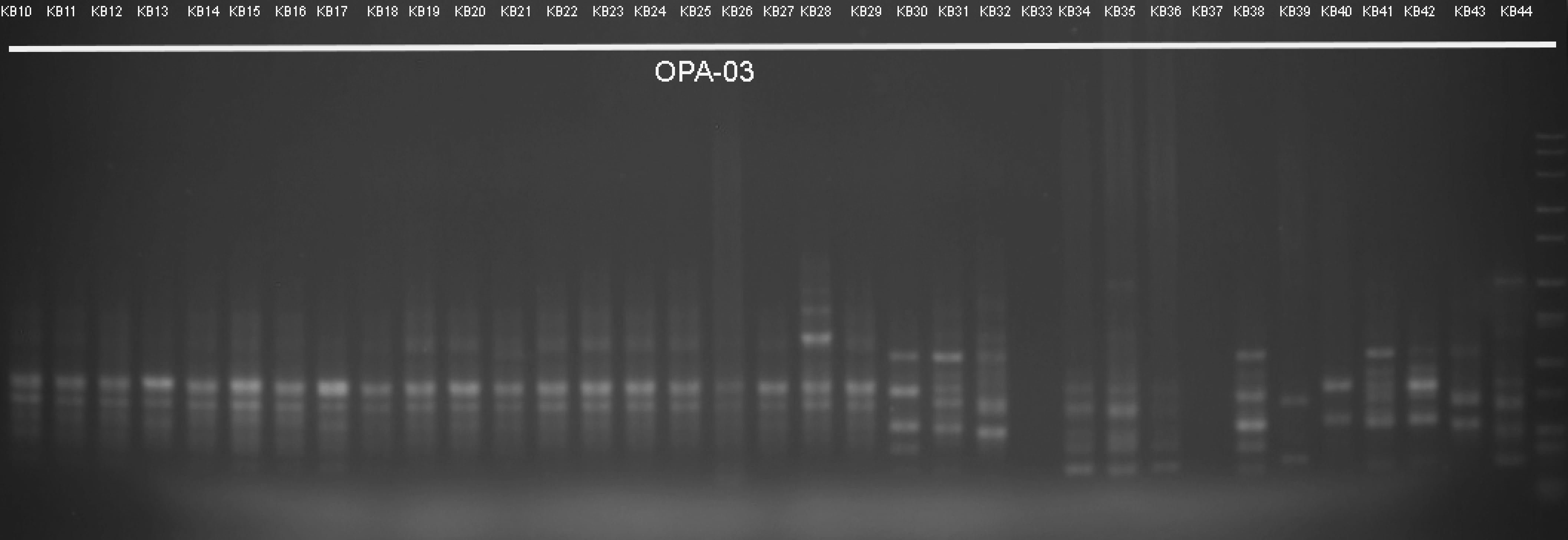
KB10 KB11 KB12 KB13 KB14 KB15 KB16 KB17 KB18 KB19 KB20 KB21 KB22 KB23 KB24 KB25 KB26 KB27 KB28 KB29 KB30 KB31 KB32 KB33 KB34 KB35 KB36 KB37 KB38 KB39 KB40 KB41 KB42 KB43 KB44
OPA-03

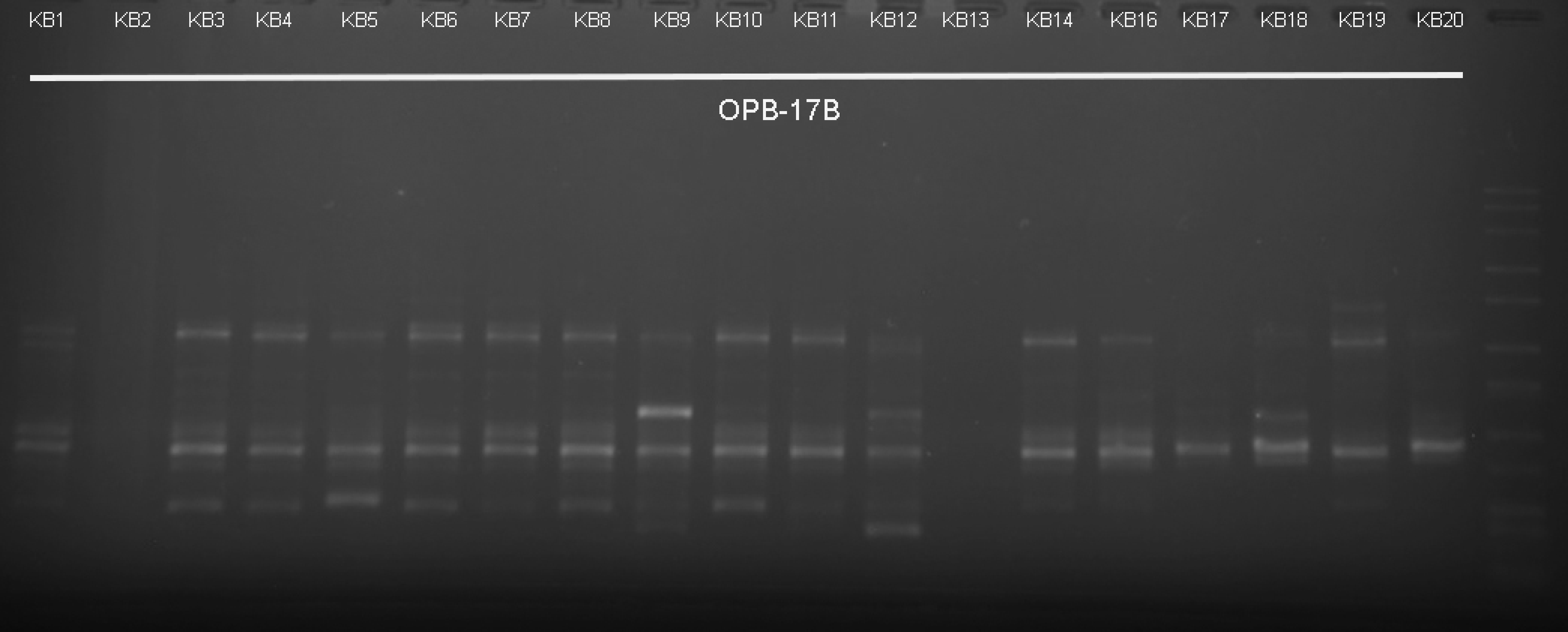
KB1 KB2 KB3 KB4 KB5 KB6 KB7 KB8 KB9 KB10 KB11 KB12 KB13 KB14 KB16 KB17 KB18 KB19 KB20
OPB-17B

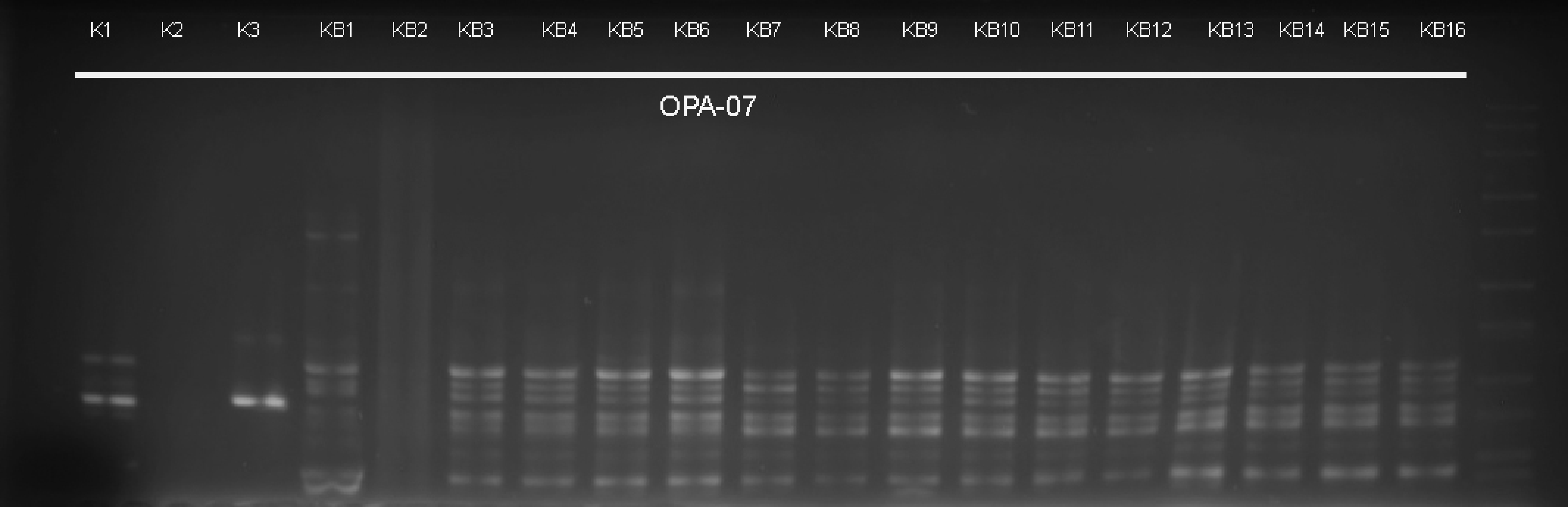
K1
K2
K3
KB1
KB2
KB3
KB4
KB5
KB6
KB7
KB8
KB9
KB10
KB11
KB12
KB13
KB14
KB15
KB16
OPA-07

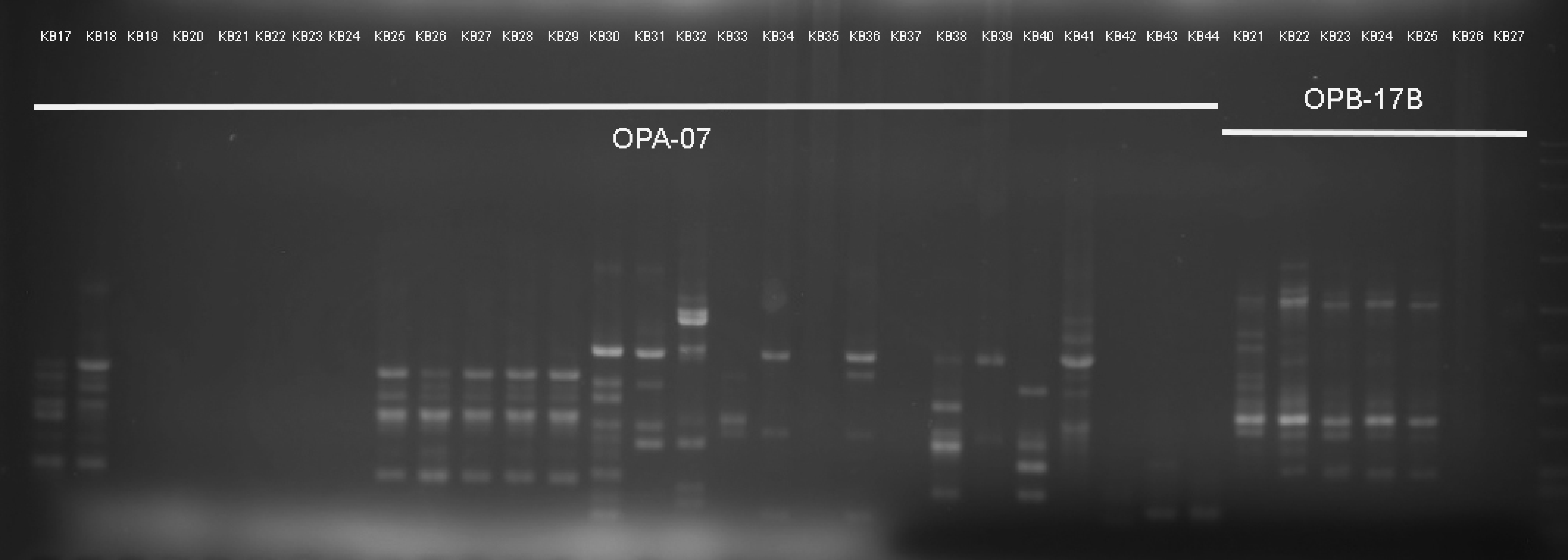
KB17 KB18 KB19 KB20 KB21 KB22 KB23 KB24 KB25 KB26 KB27 KB28 KB29 KB30 KB31 KB32 KB33 KB34 KB35 KB36 KB37 KB38 KB39 KB40 KB41 KB42 KB43 KB44 KB21 KB22 KB23 KB24 KB25 KB26 KB27
OPA-07
OPB-17B

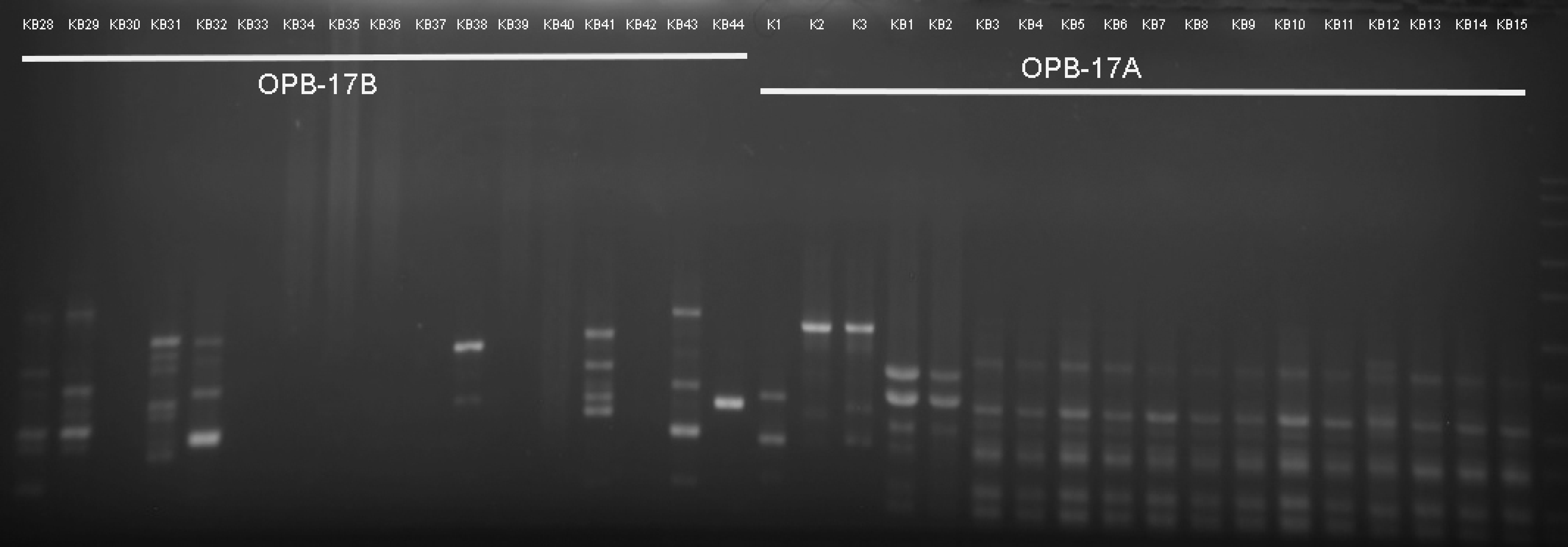
KB28 KB29 KB30 KB31 KB32 KB33 KB34 KB35 KB36 KB37 KB38 KB39 KB40 KB41 KB42 KB43 KB44
K1 K2 K3 KB1 KB2 KB3 KB4 KB5 KB6 KB7 KB8 KB9 KB10 KB11 KB12 KB13 KB14 KB15
OPB-17B
OPB-17A

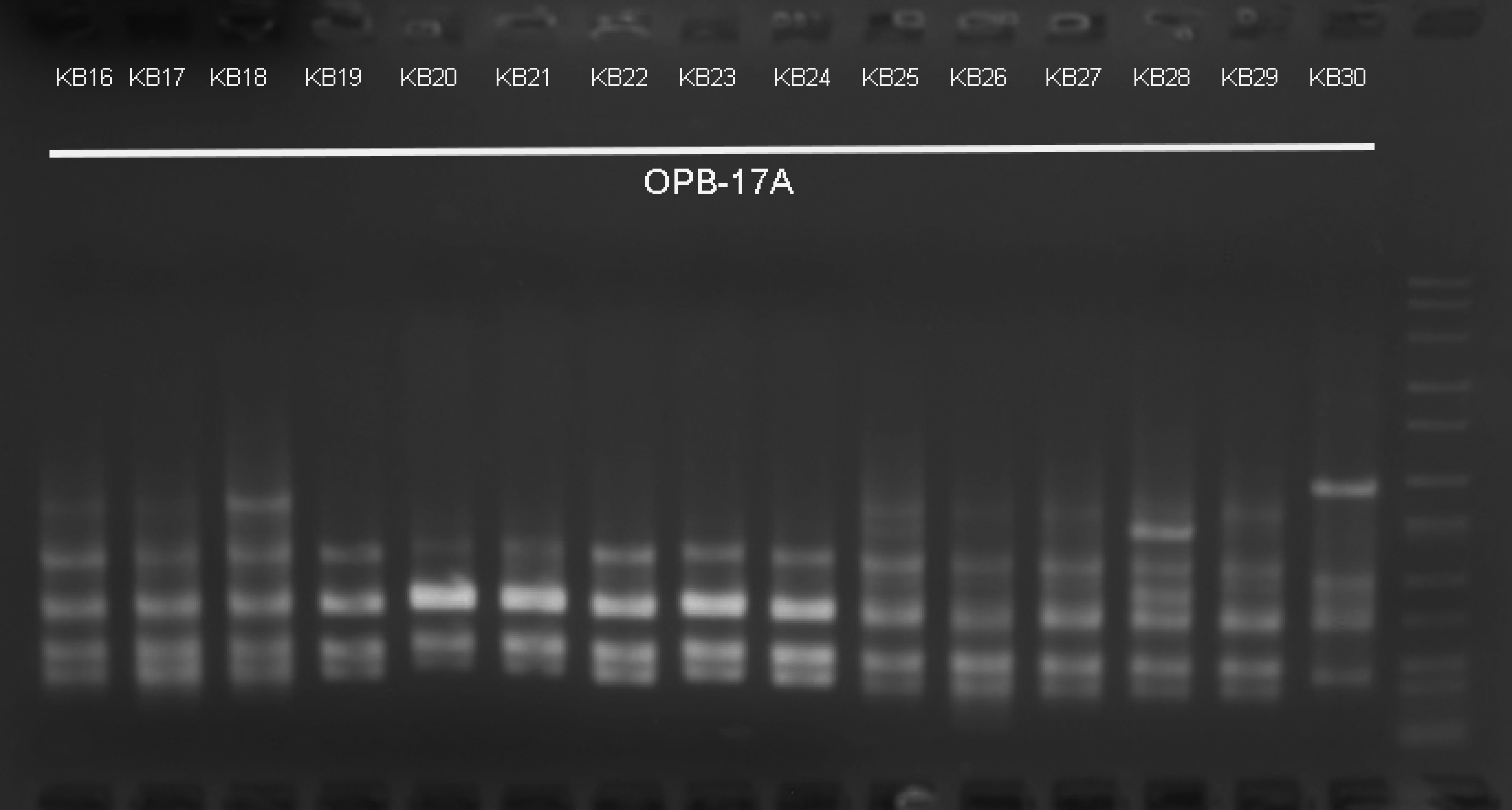
KB16 KB17 KB18 KB19 KB20 KB21 KB22 KB23 KB24 KB25 KB26 KB27 KB28 KB29 KB30
OPB-17A

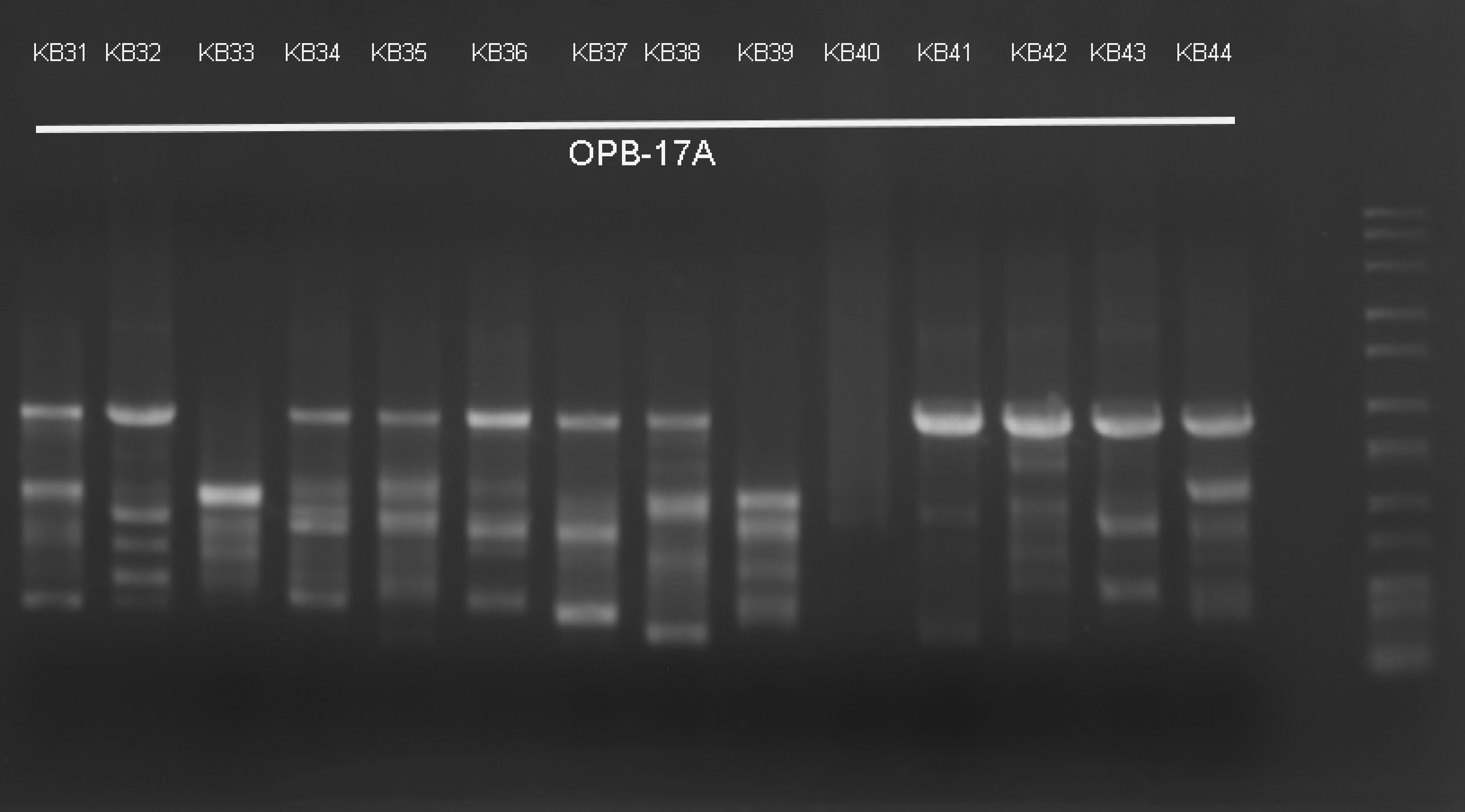
KB31 KB32 KB33 KB34 KB35 KB36 KB37 KB38 KB39 KB40 KB41 KB42 KB43 KB44
OPB-17A

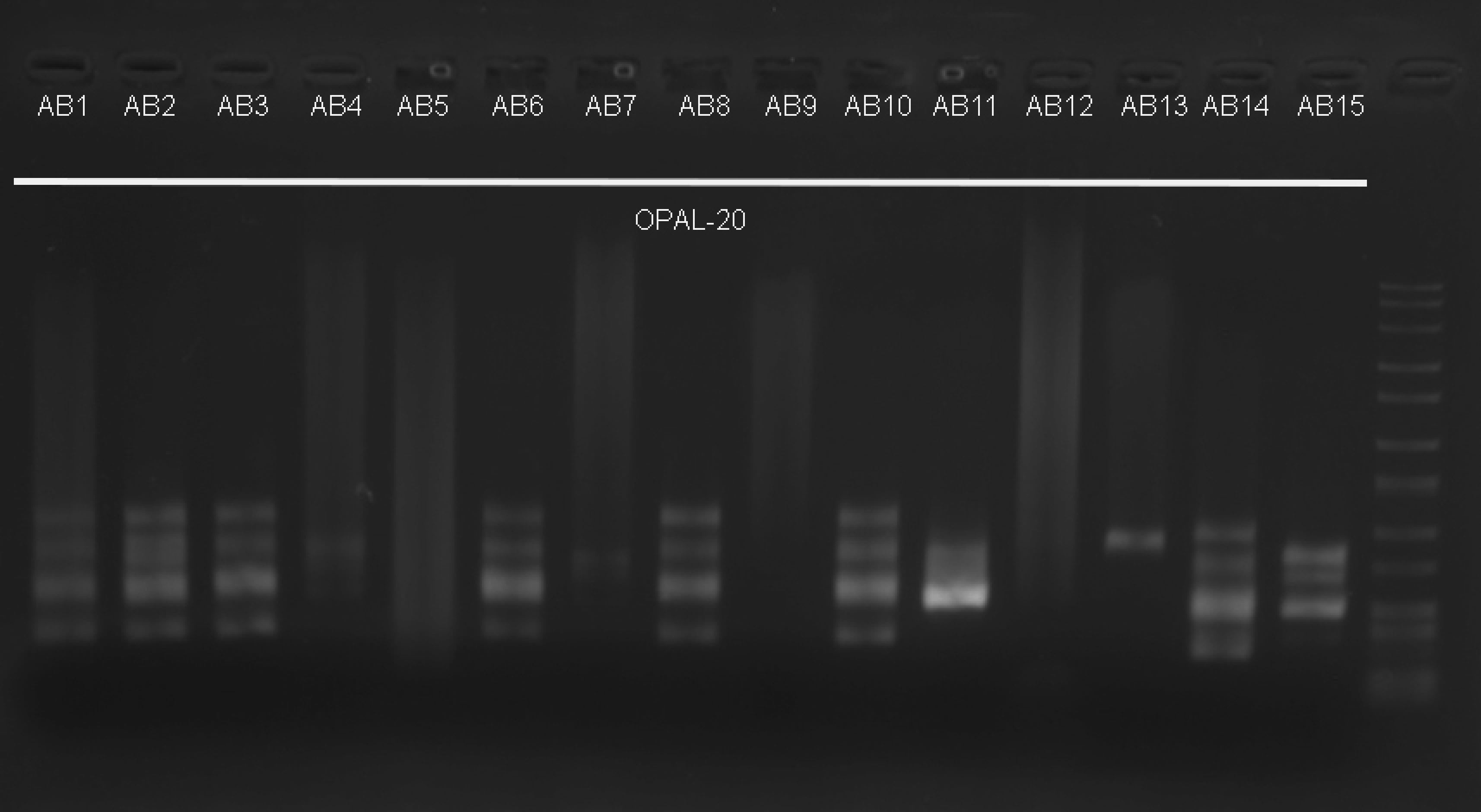
AB1
AB2
AB3
AB4
AB5
AB6
AB7
AB8
AB9
AB10
AB11
AB12
AB13
AB14
AB15
OPAL-20

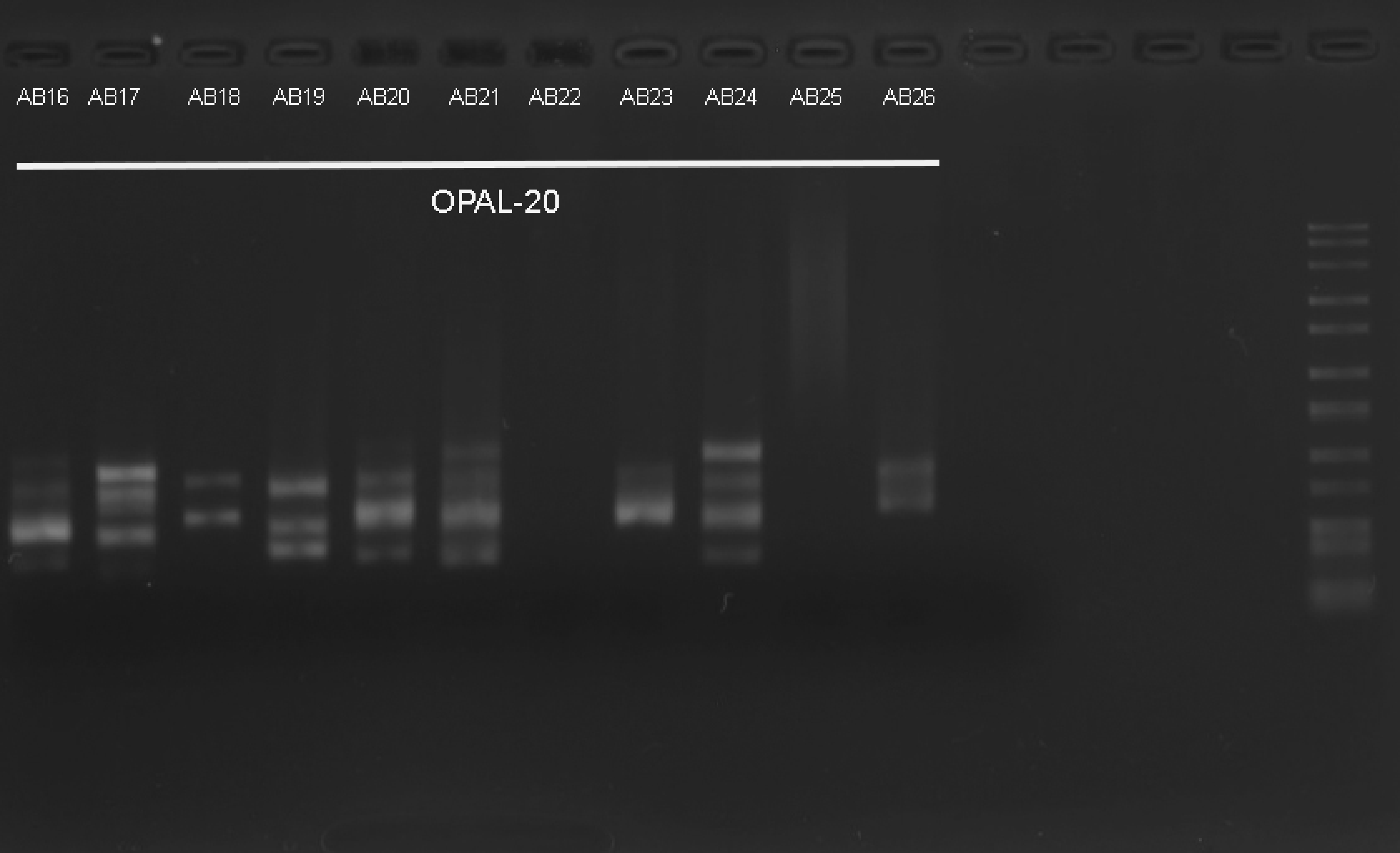
AB16 AB17 AB18 AB19 AB20 AB21 AB22 AB23 AB24 AB25 AB26
OPAL-20

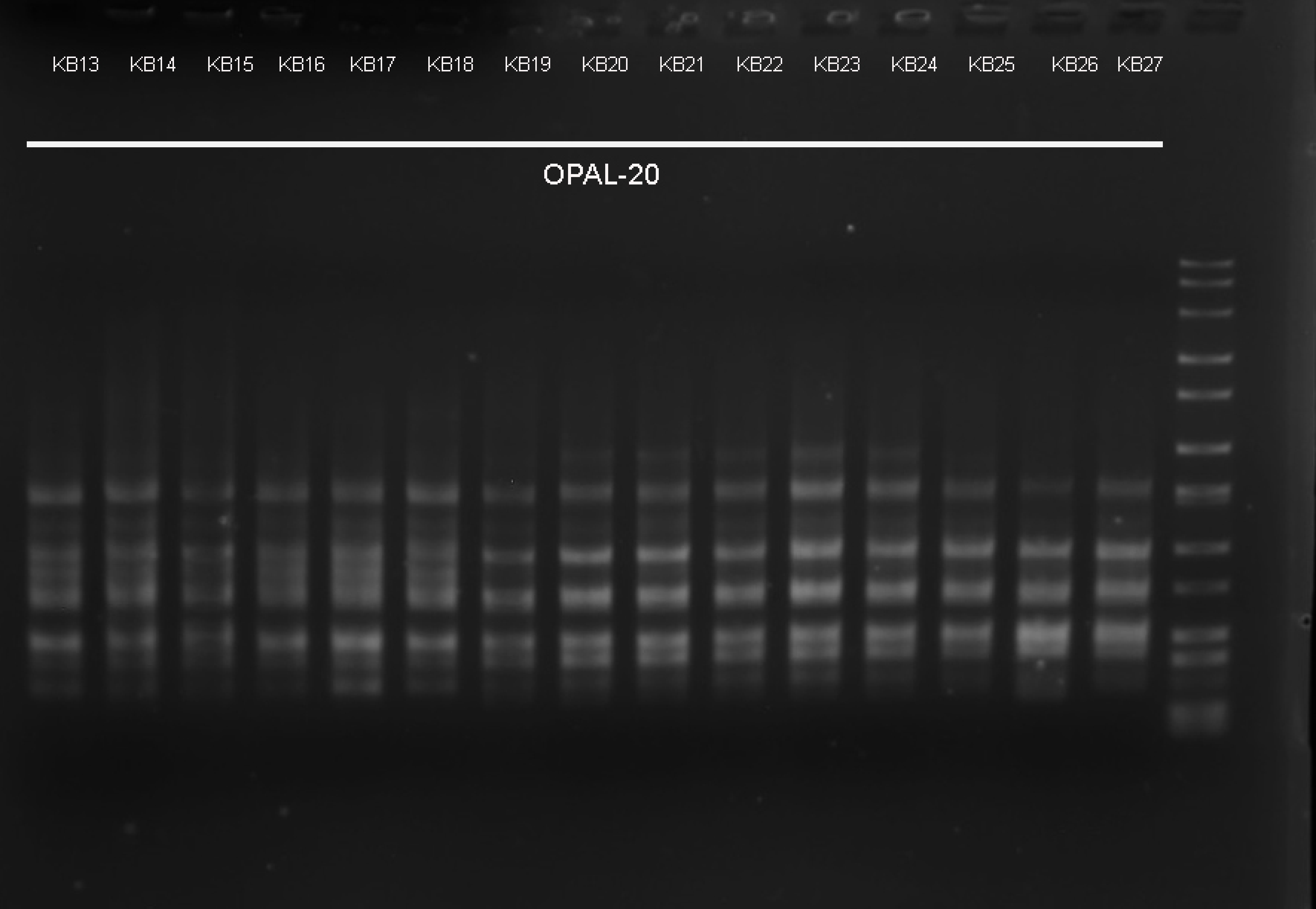
KB13 KB14 KB15 KB16 KB17 KB18 KB19 KB20 KB21 KB22 KB23 KB24 KB25 KB26 KB27
OPAL-20

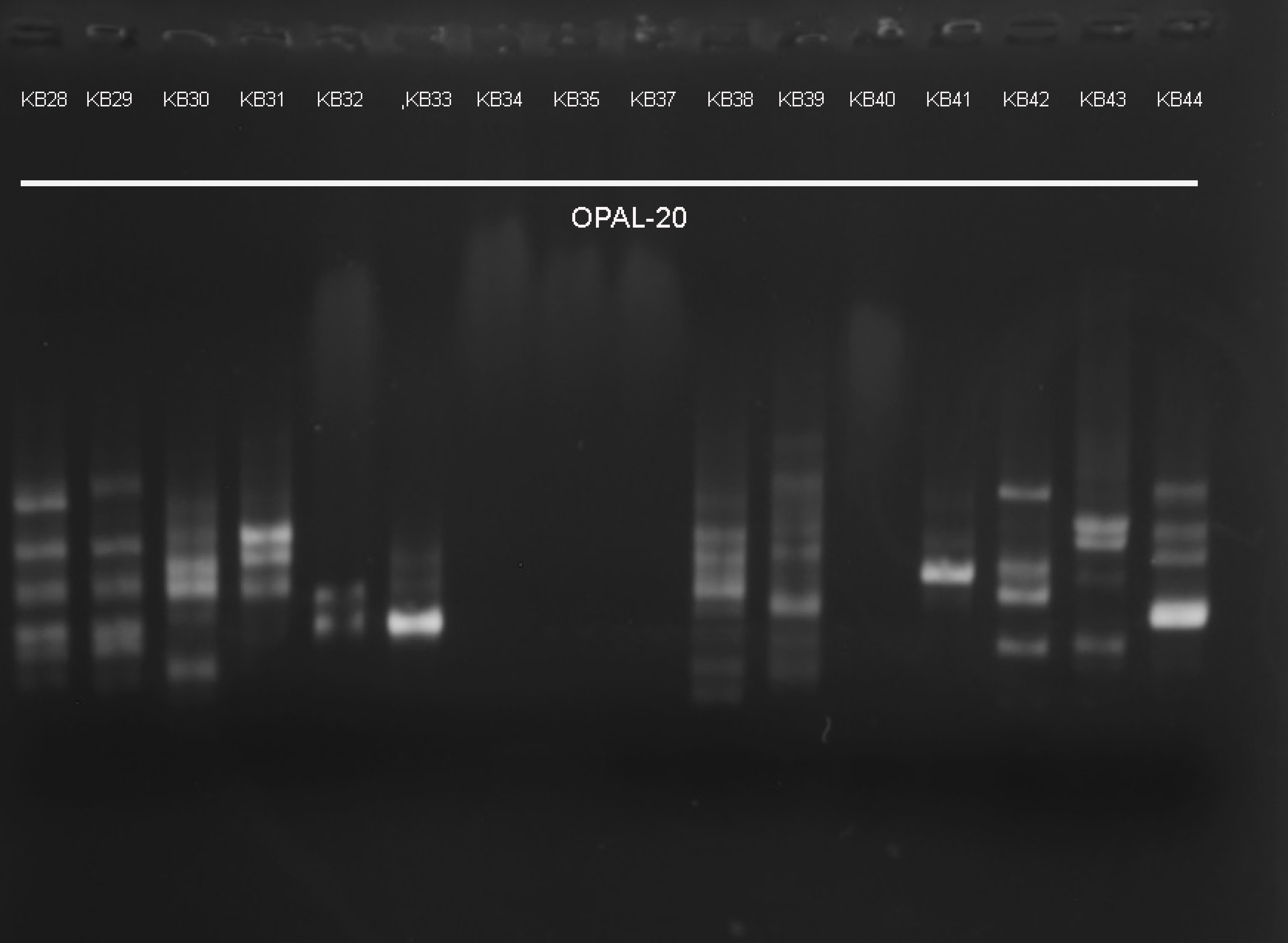
KB28 KB29 KB30 KB31 KB32 ,KB33 KB34 KB35 KB37 KB38 KB39 KB40 KB41 KB42 KB43 KB44
OPAL-20

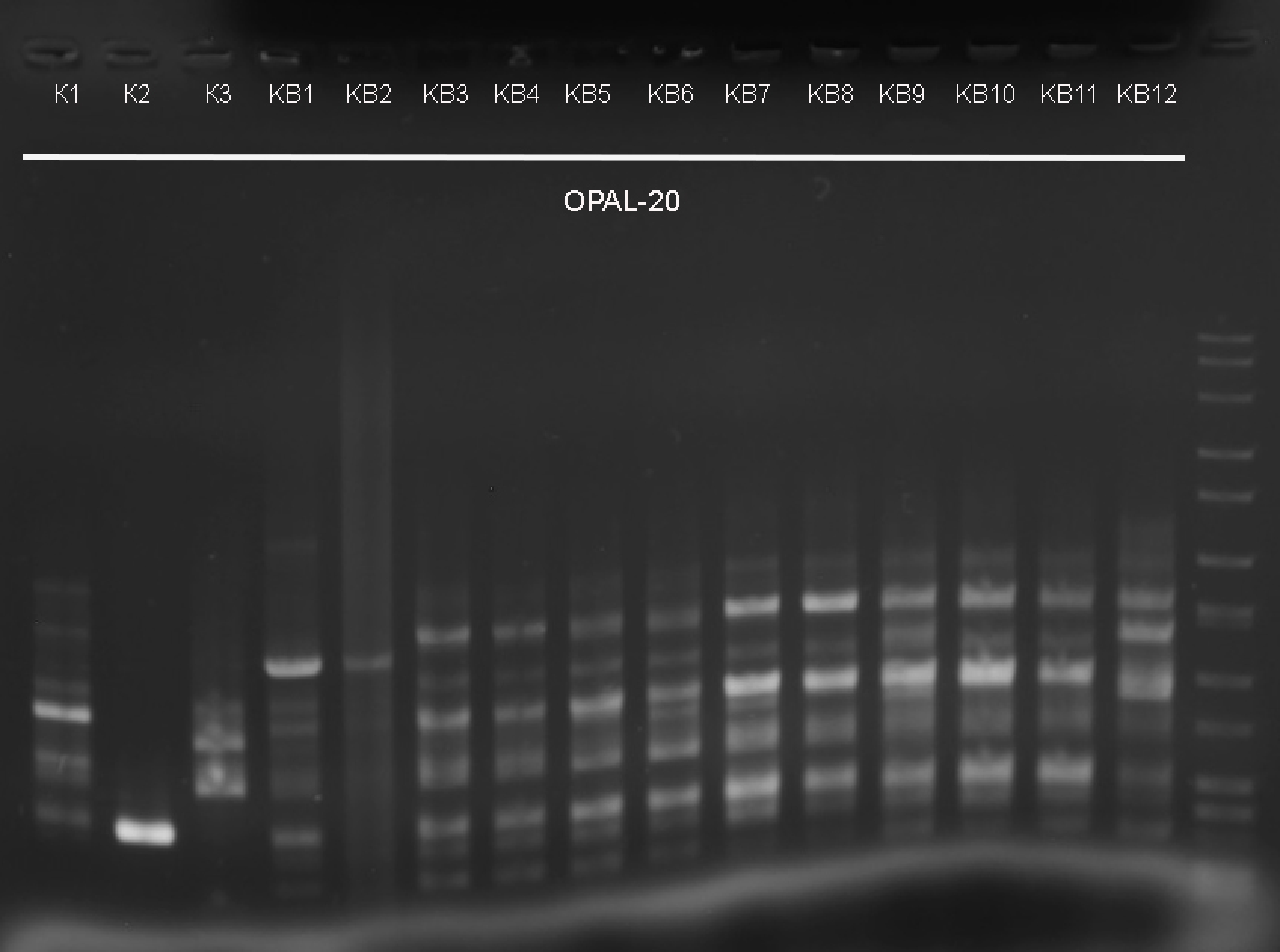
K1
K2
K3
KB1
KB2
KB3
KB4
KB5
KB6
KB7
KB8
KB9
KB10
KB11
KB12
OPAL-20

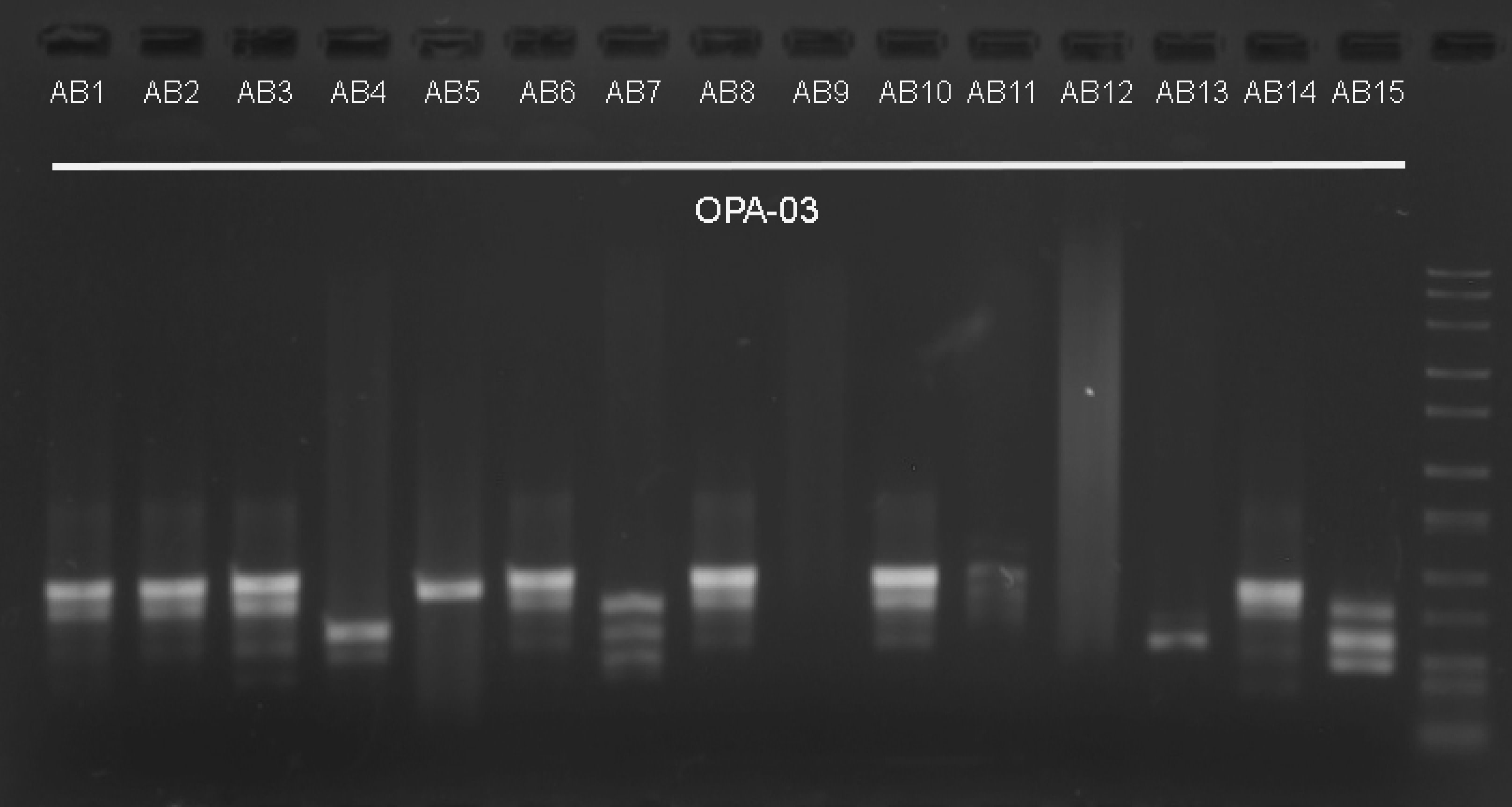
AB1 AB2 AB3 AB4 AB5 AB6 AB7 AB8 AB9 AB10 AB11 AB12 AB13 AB14 AB15
OPA-03

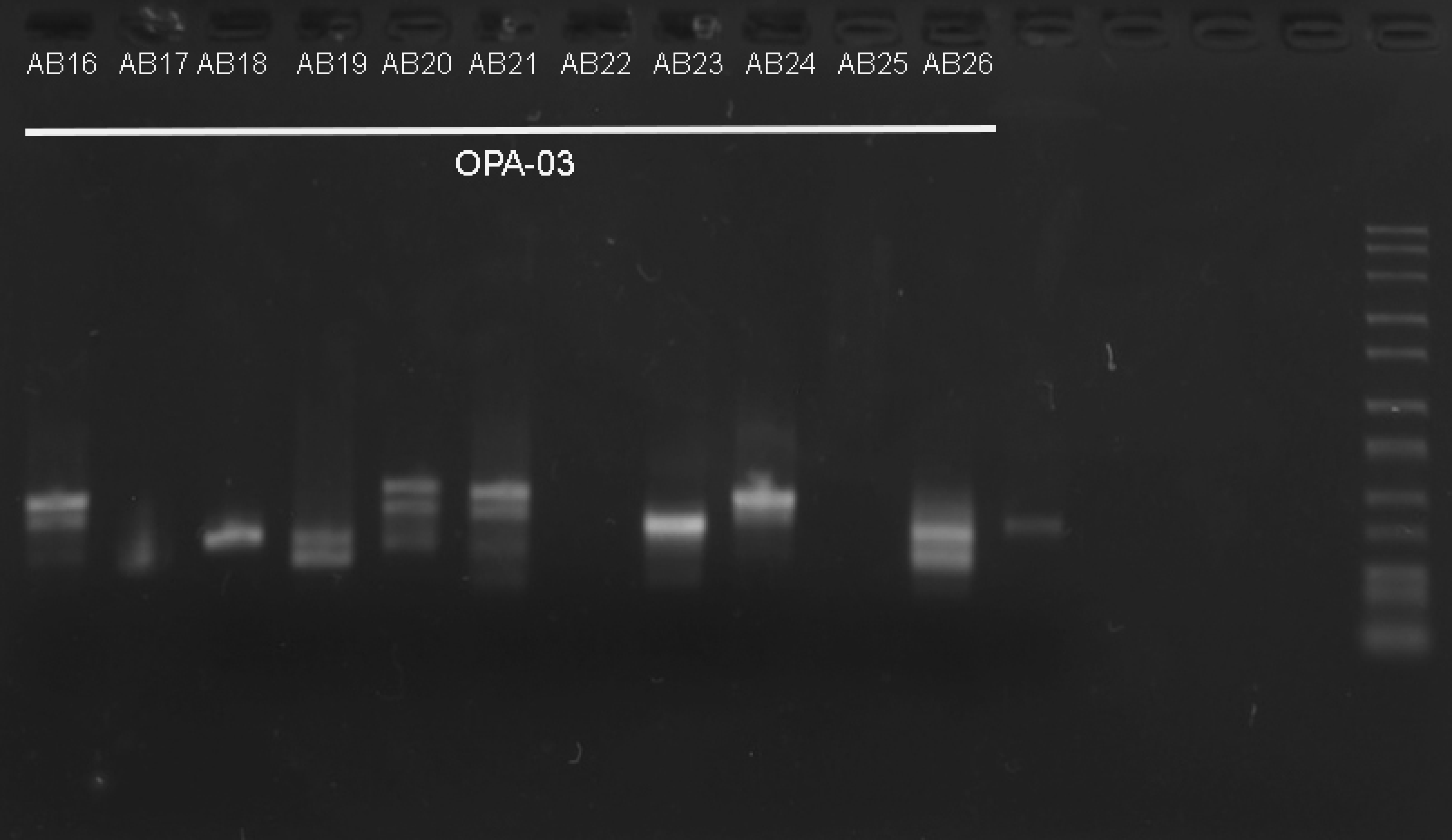
AB16 AB17 AB18 AB19 AB20 AB21 AB22 AB23 AB24 AB25 AB26
OPA-03

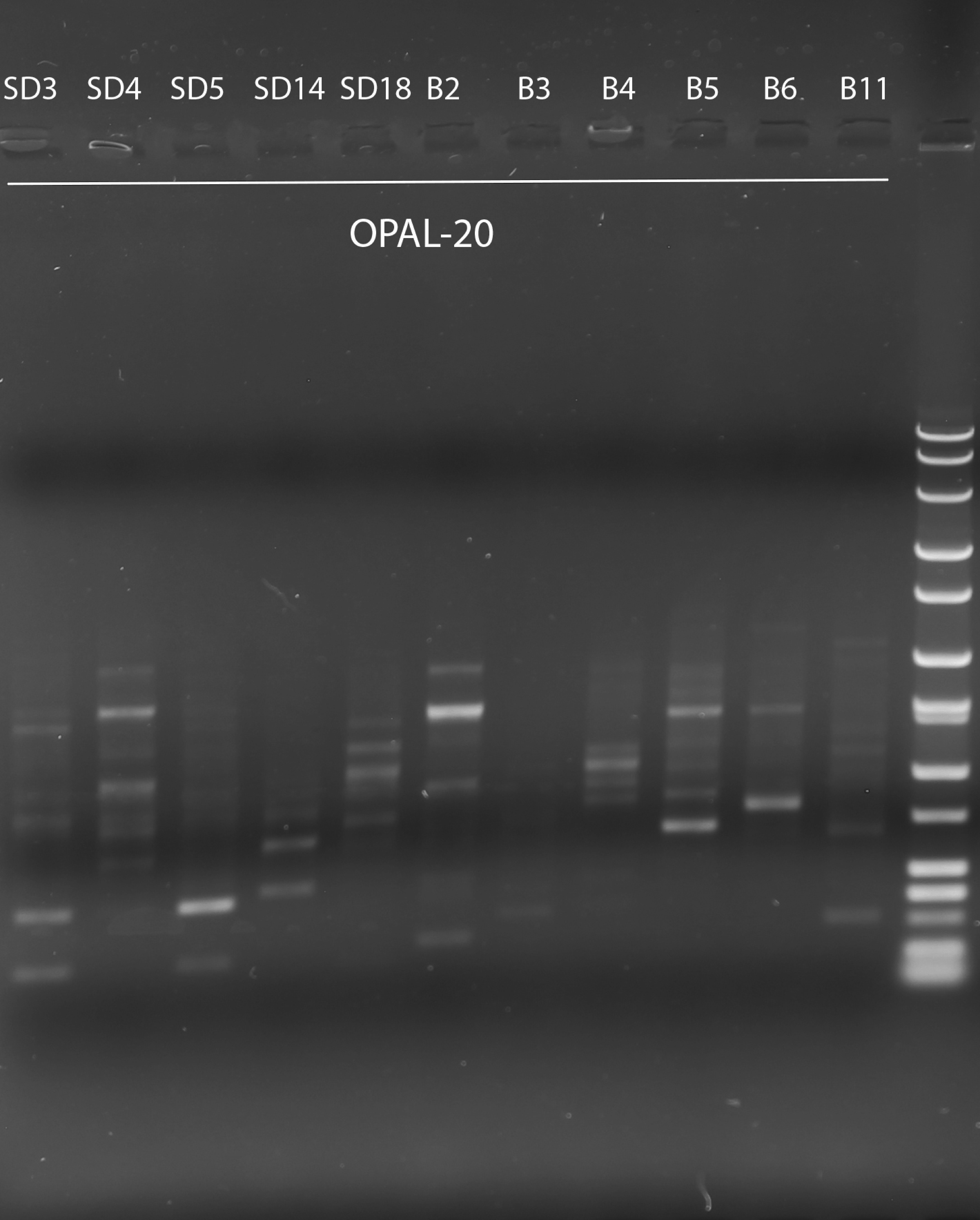
SD3 SD4 SD5 SD14 SD18 B2 B3 B4 B5 B6 B11
OPAL-20

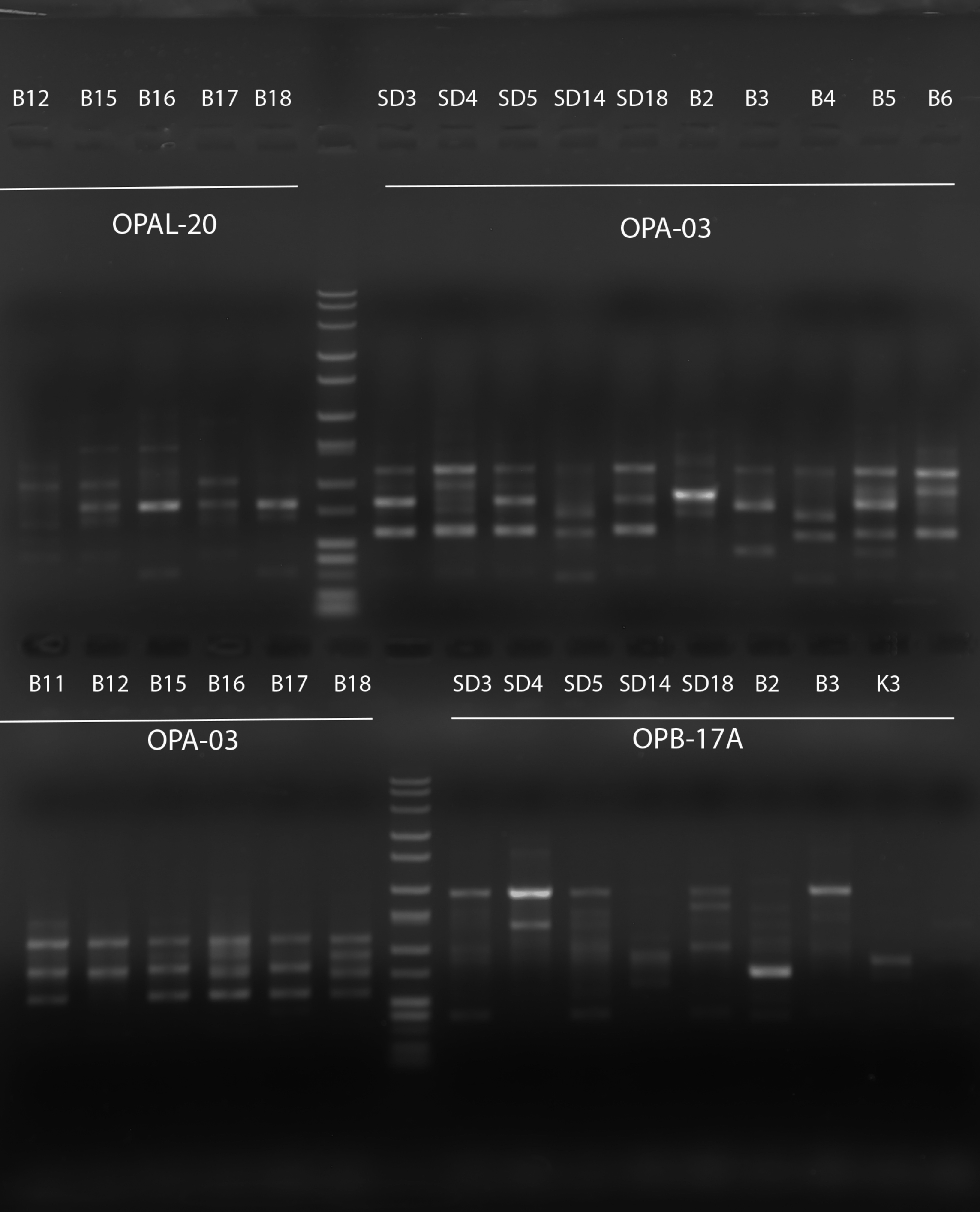
B12 B15 B16 B17 B18
SD3 SD4 SD5 SD14 SD18 B2 B3 B4 B5 B6
OPAL-20
OPA-03
B11 B12 B15 B16 B17 B18
SD3 SD4 SD5 SD14 SD18 B2 B3 K3
OPA-03
OPB-17A

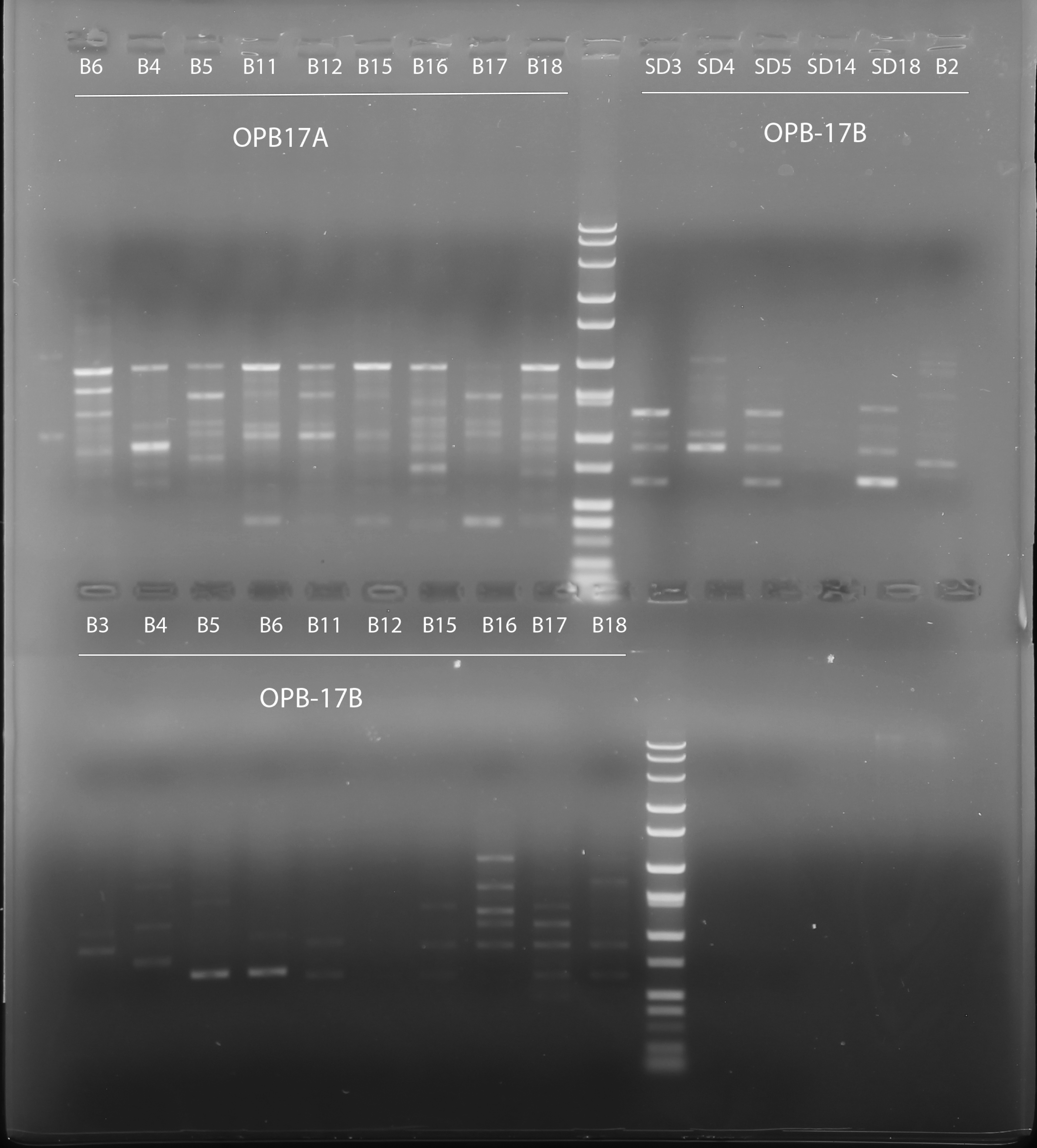
B6 B4 B5 B11 B12 B15 B16 B17 B18
OPB17A
SD3 SD4 SD5 SD14 SD18 B2
OPB-17B
B3 B4 B5 B6 B11 B12 B15 B16 B17 B18
OPB-17B

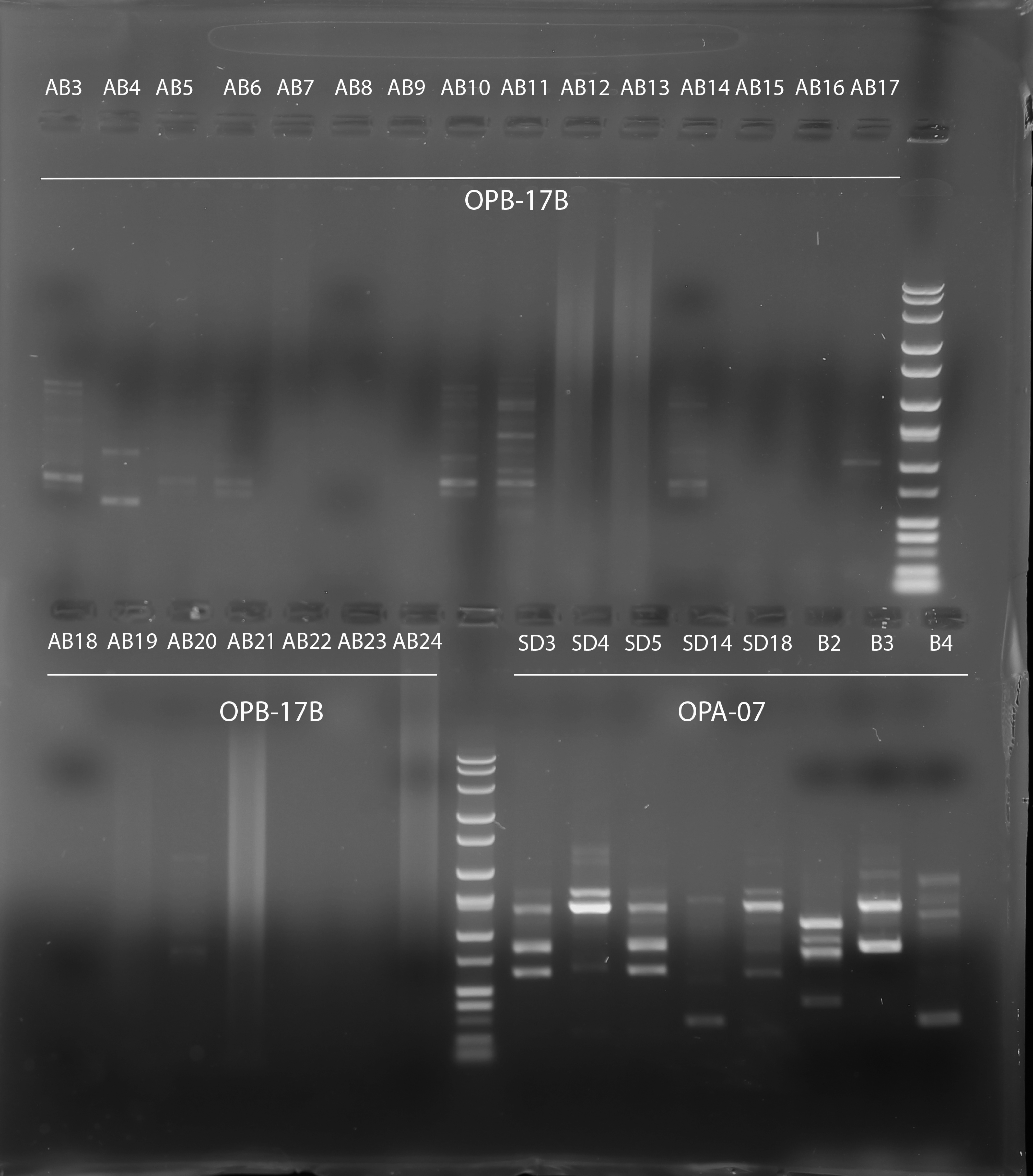
AB3 AB4 AB5 AB6 AB7 AB8 AB9 AB10 AB11 AB12 AB13 AB14 AB15 AB16 AB17
OPB-17B
AB18 AB19 AB20 AB21 AB22 AB23 AB24
SD3 SD4 SD5 SD14 SD18 B2 B3 B4
OPB-17B
OPA-07

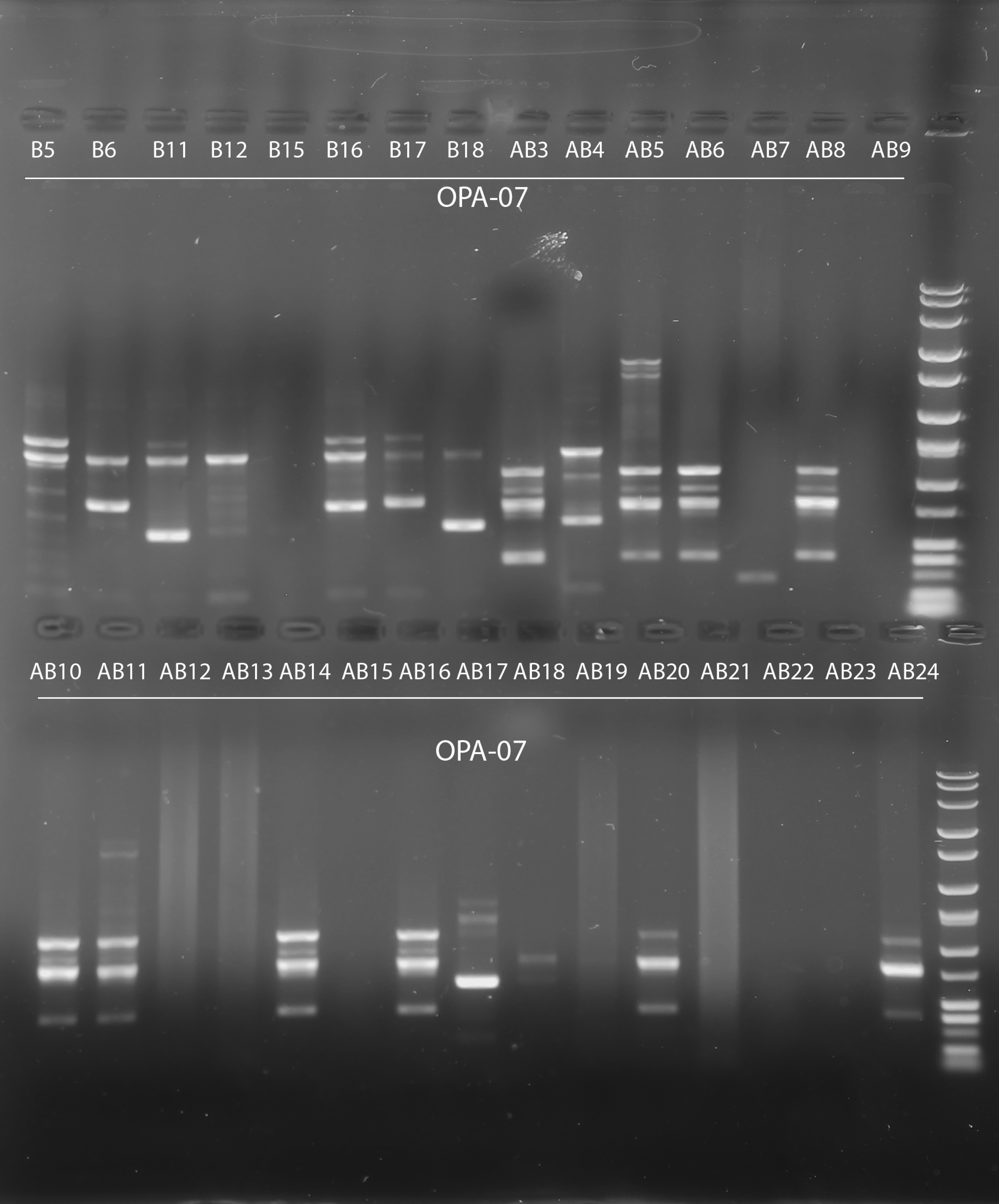
B5 B6 B11 B12 B15 B16 B17 B18 AB3 AB4 AB5 AB6 AB7 AB8 AB9
OPA-07
AB10 AB11 AB12 AB13 AB14 AB15 AB16 AB17 AB18 AB19 AB20 AB21 AB22 AB23 AB24
OPA-07
